# The Effect of EGR1 on the Proliferation of Dermal Papilla Cells

**DOI:** 10.3390/genes13071242

**Published:** 2022-07-14

**Authors:** Yeling Xu, Shanhe Wang, Xiukai Cao, Zehu Yuan, Tesfaye Getachew, Joram M. Mwacharo, Aynalem Haile, Xiaoyang Lv, Wei Sun

**Affiliations:** 1 College of Animal Science and Technology, Yangzhou University, Yangzhou 225009, China; yelingxu2001@163.com (Y.X.); shanhe12315@163.com (S.W.); 2 International Joint Research Laboratory in Universities of Jiangsu Province of China for Domestic Animal Germplasm Resources and Genetic Improvement, Yangzhou 225009, China; cxkai0909@163.com (X.C.); yuanzehu1988@163.com (Z.Y.); t.getachew@cgiar.org (T.G.); j.mwacharo@cgiar.org (J.M.M.); a.haile@cgiar.org (A.H.); 3 Joint International Research Laboratory of Agriculture and Agri-Product Safety of Ministry of Education of China, Yangzhou University, Yangzhou 225009, China; 4 International Centre for Agricultural Research in the Dry Areas, Addis Ababa 999047, Ethiopia

**Keywords:** Hu sheep, dermal papilla cells, early growth response factor 1, cell proliferation

## Abstract

Early growth response factor 1 (*EGR1*) is a zinc-finger transcription factor that plays a vital role in the development of hair follicles. According to our previous studies, *EGR1* is a transcriptional promoter of the bone morphogenetic protein 7 (*BMP7*), a candidate gene involved in the proliferation of dermal papilla cells. Since hair follicles are the basis of lambskin pattern formation and dermal papilla cells (DPCs) act on hair follicle growth, in order to elucidate the role of *EGR1* and hair follicles, this study aimed to investigate the biological role of *EGR1* in DPCs. In our study, the *EGR1* coding sequence (CDS) region was firstly cloned by polymerase chain reaction, and bioinformatics analysis was performed. Then, the function of *EGR1* was detected by 5-ethynyl-2’-deoxyuridine (EDU) and Cell Counting Kit-8 (CCK8), and Western blot (WB) was conducted to analyze the cellular effect of *EGR1* on DPCs. The proliferative effect of *EGR1* on DPCs was also further confirmed by detecting its expression by qPCR and WB on marker genes of proliferation, including *PCNA* and *CDK2*. The sequence of the *EGR1* CDS region of a lamb was successfully cloned, and its nucleic acid sequence was analyzed and found to be highly homologous to *Rattus norvegicus*, *Mus musculus*, *Bos taurus* and *Homo sapiens*. Predictive analysis of the protein encoded by *EGR1* revealed that it is an extra-membrane protein, and not a secretory protein, with subcellular localization in the nucleus and cytoplasm. The proliferative effect of DPCs was significantly stronger (*p* < 0.01) in *EGR1* up-regulated DPCs compared to the controls, while the opposite result was observed in *EGR1* down-regulated DPCs. Markers of proliferation including *PCNA* and *CDK2* also appeared to be differentially upregulated in *EGR1* gene overexpression compared to the controls, with the opposite result in *EGR1* gene downregulation. In summary, our study revealed that *EGR1* promotes the proliferation of DPCs, and we speculate that *EGR1* may be closely associated with hair follicle growth and development.

## 1. Introduction

**Figure 1:**
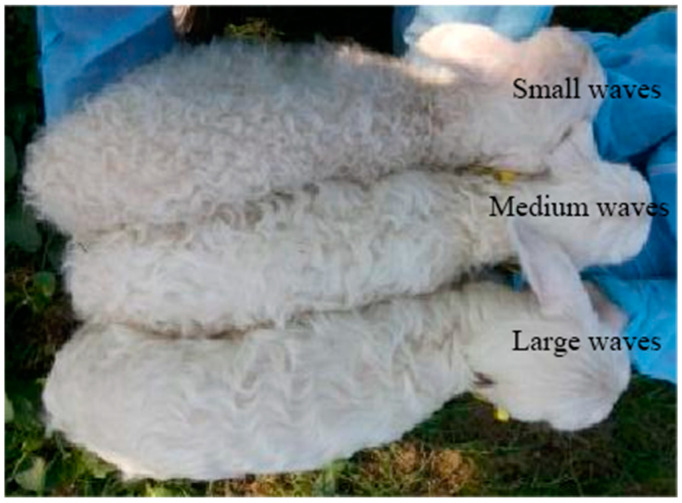
The different patterns of lambskin of Hu lambs.

Hu sheep are famous for their white lambskin with a wavy pattern, which is generally classified into three types—small waves, medium waves, and large waves—determining the Hu lambskin quality (Figure 1). It is usually considered that small waves indicate the best quality [1]. With the continuous growth of mutton consumption, livestock producers have been more concerned with meat and reproductive traits, thus neglecting lambskin traits, resulting in the gradual decline of lambskin quality in the selection and breeding process, and the high-quality germplasm resources are threatened.

The type of lambskin pattern is determined by the different curling degree of the wool, which is influenced by the growth and development of the hair follicles. The hair follicle can be divided into epidermal and dermal components from a developmental biology perspective. Hair follicles are complex in composition, and include the epidermal component and the dermis. In particular, the dermis consists mainly of the dermal papilla (DP) [2]. Dermal papilla cells express exocrine signaling such as Wnts, FGF and Noggin in vivo, which can act on hair follicle biological activity [3,4]. Additionally, Nissiove et al. [5] proposed a “multiple papillary centres” (MPC) hypothesis, which suggests that dermal papilla play a positive role in hair follicle development and hair growth, and that each papillary structure acts independently, leading to differences in the growth rate of hair follicle cells and thus to curved hair growth. Therefore, we believe that the dermal papilla cells are important cells affecting the pattern formation of lambskin.

Early growth response factor 1 (*EGR1*) is a zinc-finger structure that is associated with diverse cellular functions, such as proliferation, apoptosis, and migration [6,7]. Activated *EGR1* are involved in growth factors, inflammatory factors, reactive oxygen species, etc. [8]. The Ras-RAF-MEK1/2-ERK1/2 signaling pathway allows for successful transcription of *EGR1*, and *EGR1* participate in target gene transcriptional regulation to regulate their expression [9]. Numerous studies have shown that the *EGR1* gene is closely related to tumors [10], stomach cancer [11], glioma [12] and melanoma [13]; however, little is known about the direct effect of *EGR1* on dermal papilla cells. Nevertheless, *EGR1* was found to be an important transcription factor in the bone morphogenetic protein 7 (*BMP7*) promoter region, and *BMP7* has been proved to be a candidate gene in the proliferation of dermal papilla cells, collectively. We speculated that *EGR1* may also affect the proliferation of dermal papilla cells, which initially affects the pattern formation of lambskin [14]. In summary, we suggested that the important transcription factor *EGR1* of *BMP7* could regulate the proliferation of dermal papilla cells of Hu sheep, which in turn affects wool bending growth, leading to lambskin pattern formation.

To explore the biological role of *EGR1* in DPCs, we cloned the sequence of the *EGR1* CDS region and a series of bioinformatics analyses were conducted. The role of *EGR1* in dermal papilla cells was further investigated by examining the tissue expression profile of *EGR1*, and the effects of *EGR1* overexpression and interference on cells, thus speculating on the potential role of *EGR1* in dermal follicle growth. Our results can provide the scientific basis for analyzing the molecular mechanism of lambskin pattern formation and pave the way for subsequent studies.

## 2. Materials and Methods

### 2.1. Experimental Animals

All lambs used in this experiment were from the Suzhou Stud Farm (Suzhou, China). A total of three pairs of full sibling individuals, including one straight hair and one small waves per pair. We collected approximately 0.8 cm^2^ of skin tissue from the same dorsal side of 6 three-day-old Hu lambs, which were rapidly stored in RNA preservation solution (TAKARA, Dalian, China). The hair follicles, heart, liver, spleen, lung, kidney, and muscle used in the tissue expression profile were obtained from laboratory preservation. Total RNA was extracted using Trizol (TIANGEN, Beijing, China). The first strand of cDNA was prepared using the FastKing gDNA Dispelling RT SuperMix (TIANGEN, Beijing, China), referring to the product instructions, and then stored at −20 °C.

Our experimental protocol was approved by the Animal Ethics Committee of Yangzhou University (No: NSFC2020-NFY-1).

### 2.2. Cloning of the EGR1 CDS Region

The primers were designed to amplify the *EGR1* CDS sequence (1632 bp, GenBank accession number: EU552504.1) from the sheep *EGR1* sequence provided by NCBI. The enzyme digestion sites were selected with EcoR I (G^AATTC) and Hind I III (A^AGCTT). The primer sequences were:

*EGR1*-F1: CTAGCGTTTAAACTTAAGCTTATGGCGGCAGCCAAGGC (5′→3′)

*EGR1*-R1: TGCTGGATATCTGCAGAATTCTTAGCAATTTCAATTGTCCTGGGA (5′→3′)

PCR amplification was performed according to the instructions of PrimeSTAR^®^ Max DNA Polymerase (Takara Bio, Beijing, China), using cDNA as a template. We used 50 μL of PCR amplification system: PrimeSTAR Max Premix (2X) 25 μL, *EGR1*-F1 1 μL, *EGR1*-R1 1 μL, cDNA 2 μL, and ddH2O 21 μL. Reaction conditions: denaturation at 98 °C for 10 s, annealing at 60 °C for 15 s, extension at 72 °C for 90 s, 40 cycles. The products were identified by 1% agarose gel electrophoresis.

### 2.3. Bioinformatics Analysis of EGR1

According to the amino acid sequences of Hu sheep and other different species, the phylogenetic tree was calculated according to the adjacency method using Mega 11 software, including *Homo sapiens* (MK681487.1) (hsa), *Bos taurus* (AY924307.1) (bta), *Mus musculus* (BC138615.1) (mmu), and *Rattus norvegicus* (AY551092.1) (rno).

The following were conducted: analysis of basic protein chemistry using the ProtParam tool (https://web.expasy.org/protparam/, accessed on 3 April 2022); potential signal peptide cleavage site prediction using SignalP4.1 (https://services.healthtech.dtu.dk/service.php?SignalP-4.1, accessed on 3 April 2022); analysis of glycosylation sites using NetOGlyc4.0 (https://services.healthtech.dtu.dk/service.php?NetOGlyc-4.0, accessed on 3 April 2022); prediction of phosphorylation sites of amino acid sequences using NetPhos3.1 (https://services.healthtech.dtu.dk/service.php?NetPhos-3.1, accessed on 3 April 2022); prediction of conserved structural domains of amino acid sequences using SMART (http://smart.embl-heidelberg.de/, accessed on 3 April 2022); prediction of secondary structure using ProtScale (https://web.expasy.org/protscale/, accessed on 3 April 2022) to analyze the hydrophilicity of amino acid sequences; GOR IV (https://npsa-prabi.ibcp.fr/cgi-bin/npsa_automat.pl?page=npsa_gor4.html, accessed on 3 April 2022) to predict the secondary structure of proteins; transmembrane analysis of protein sequences using TMHMM-2.0 (https://services.healthtech.dtu.dk/service.php?TMHMM-2.0, accessed on 3 April 2022); and analysis of protein subcellular localization using PSORT II (https://psort.hgc.jp/form2.html, accessed on 3 April 2022).

### 2.4. Overexpression Vector Construction and siRNA Synthesis

The cloned product of the *EGR1* CDS region was purified and ligated to the pcDNA3.1(+) vector. Then, recombinant vectors were sent to Qingke Biotechnology Co., Ltd. (Nanjing, China) for verification. The successful vector was named pcDNA3.1-EGR1. The siRNA of *EGR1* was designed and synthesized by Suzhou GenePharma Co., Ltd. (Suzhou, China) (Table 1).

### 2.5. Cell Culture

DPCs were cultured in DMEM/F12 (HyClone, Logan, UT, USA) supplemented with 10% fetal bovine serum (Gibco, Grand Island, NY, USA) and 1% penicillin–streptomycin with 5% CO_2_ at 37 °C [15].

pcDNA3.1-EGR1 and the siRNA were introduced into DPCs. After that, DPCs were collected at 48 h post-transfection for subsequent studies, including negative controls (NC).

### 2.6. Cell Proliferation

Prior to cell proliferation assays, DPCs were transferred to 96-well plates. Then, 10 µL of Cell Counting Kit (CCK-8) was added to each well under the time period of 0, 24, 48 and 72 h. The Tecan Infinite F200/M200 microplate instrument (Tecan, Männedorf, Switzerland) was used to detect OD value at 450 nm. Additionally, EDU immunofluorescence was used to further detect proliferating cells.

### 2.7. RT-qPCR

The SYBR Green qPCR kit (Takara, Dalian, China) was used to detect the mRNA expression levels of the above reverse transcription products. To further validate the effect of this gene of *EGR1* on dermal papilla cells, we also used proliferation-related markers, including proliferating cell nuclear antigen (*PCNA*), and cyclin-dependent kinase 2 (*CDK2*). According to the sequences information in GenBank, primers were designed and synthesized by Qingke Biotechnology Co., Ltd. (Nanjing, China). We used the house-keeping gene (*GAPDH*) as an internal reference. All treatments included three biological replicates, and the primers information is shown in Table 2.

### 2.8. Western Blot

After 48 h of transfection, the DPCs proteins were disposed of with RIPA lysis buffer (Beyotime, Shanghai, China), and concentrations were detected using the BCA method. The proteins were separated and then transferred to PVDF membranes, which were probed with 1:500 rabbit anti-EGR1 (Affinity, Melbourne, Australia), 1:2500 mouse anti-GAPDH (ABclonal, Wuhan, China), 1:1000 rabbit anti-PCNA (Abcam, Cambridge, UK), 1:1000 rabbit anti-CDK2 (Abcam, Cambridge, UK), 1:3000 goat anti-rabbit IgG HRG antibody (ABclon, Wuhan, China), and 1:3000 goat anti-mouse (ABclonal, Wuhan, China). The protein expressions were measured using the ECL Western Blot kit (BioSharp, Hefei, China), and analysed by the ChemiDoc^TM^ Analysis System (Bio-Rad, Hercules, CA, USA).

### 2.9. Analysis of EGR1 Expression Profiles in Different Tissues

RNA from various tissues of Hu sheep was used, reverse transcribed, and then RT-qPCR was carried out to detect the mRNA expression level of *EGR1* in the heart, liver, spleen, lung, kidney, muscle, and hair follicle. We used an amplification system of 20 μL: 2×TSINGKE^®^ Master qPCR Mix 10 μL, *EGR1*-F 1 μL, *EGR1*-R 1 μL, cDNA 1 μL, ddH_2_O 7 μL. Reaction procedure: 95 °C 1 min; 95 °C 10 s, 60 °C 10 s, 72 °C 10 s, total 40 cycles.

### 2.10. Statistical Analysis

All the above experiments were processed using the 2^−∆∆CT^ method [16]. Statistical analyses were performed using SPSS 26.0 software. Independent samples t-test and ANOVA were used for variance and significance testing. All experimental data were expressed as mean ± standard error (SEM).

## 3. Results

### 3.1. Cloning of the EGR1 CDS Region

According to the previously designed specific primers and the cDNA of the small waves as a template, the EGR1 CDS region was amplified. After gel electrophoresis, the amplified product was observed using a UV gel imager as a clear band around 1632 bp with good specificity, and the sequencing results were consistent with the expected results.

### 3.2. Bioinformatics Analysis of EGR1

**Figure 2:**
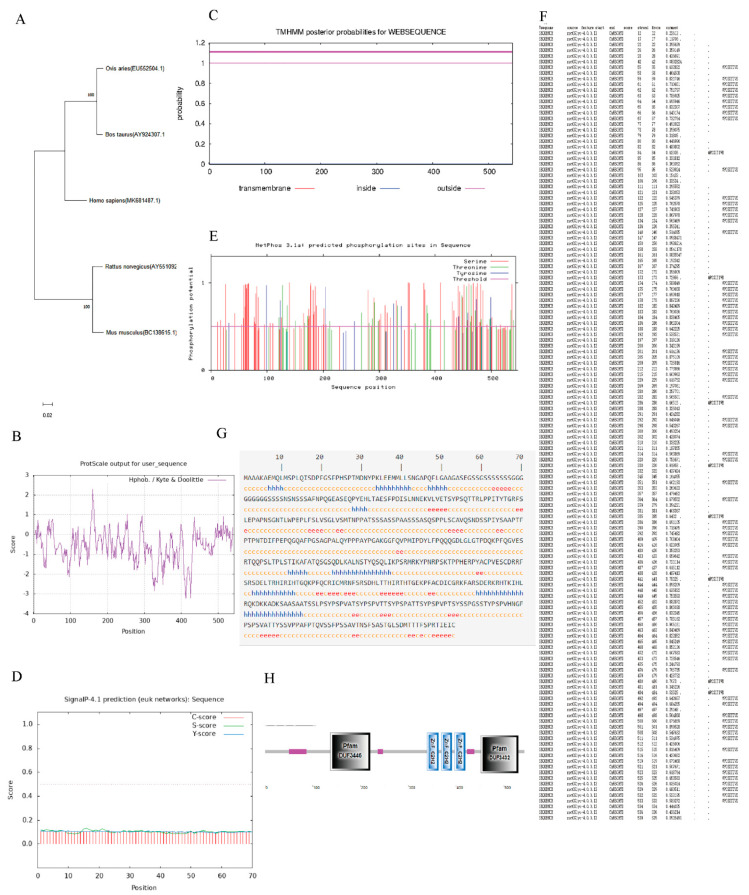
Bioinformatics analysis of *EGR1*. (**A**) Phylogenetic tree of *EGR1* between different species. (**B**) The hydropathy profile of EGR1 amino acid. (**C**) EGR1 protein transmembrane regional analyses. (**D**) EGR1 signal peptide prediction. (**E**) EGR1 phosphorylation site analyses. (**F**) EGR1 glycosyl site analyses. (**G**) EGR1 protein secondary structure prediction. (**H**) EGR1 conservative structure domain analyses.

The CDS nucleotide sequence of the sheep *EGR1* gene (EU552504.1) was compared with that of *bta*, *hsa*, *rno* and *mmu* using BLAST in NCBI, and the results showed that the homology between sheep and *bta hsa rno* and *mmu* was 97.06%, 92.03%, 84.85% and 84.70% respectively (Table 3). This result demonstrated that the gene is relatively conserved during biological evolution. A nucleotide phylogenetic tree model was constructed using MEGA11, and it was found that the sheep were most closely related to *bta* during the evolution of the species (Figure 2A).

As previously mentioned, bioinformatics analysis revealed that the *EGR1* CDS region of Hu sheep is 1632 bp and encodes 543 amino acids. The relative molecular mass is 57,512.70 Da and the isoelectric point is 8.50. In mammalian red blood cells, the protein has a half-life of 30 h and an instability coefficient of 75.20, indicating that the protein is unstable. The lipolysis index was 49.32 and the total average hydrophilicity (GRAVY) was −0.572, indicating that the protein is hydrophilic. Further hydrophobic analysis of the protein according to the ProtScale online software showed that the hydrophobic region was above the vertical coordinate of 0 (the higher the score, the stronger the hydrophobicity), and the hydrophilic region was below 0 (the lower the score, the stronger the hydrophilicity). This shows that the amino acid sequence has more hydrophilic residues than hydrophobic residues, so the overall performance is hydrophilic, which is the same as the hydrophilic result predicted by the ProtParam software (Figure 2B). The TMHMM-2.0 online software predicted the transmembrane structure of the EGR1 protein. The structure showed that all amino acids of the protein are outside the membrane (Figure 2C) and there is no transmembrane structure, so the protein is presumed to be an extramembrane protein. Prediction of the signal peptide of the EGR1 protein by the online software SignalP4.1 showed that no signal peptide sequence exists for this protein, which means that it is not a secreted protein (Figure 2D). Subcellular localization analysis using PSORT II prediction showed that the protein was localised to the nucleus and cytoplasm, with 95.7% and 4.3% of the protein, respectively. NetPhos 3.1 was used to predict the phosphorylation sites of the protein’s amino acid sequence, and these results showed that there were 115 potential phosphorylation sites, most of which were serine (Figure 2E). NetOGlyc 4.0 was used to predict the glycosylation sites of the amino acid sequence of the protein, and the results showed that 86 glycosylation sites were present (Figure 2F). Using GOR IV to analyze the EGR1 protein secondary structure (Figure 2G), it was found that 14.73% of the protein was in the alpha helix (h), 12.52% was in the extended chain (e), and 72.74% was in the random coiled coil (c). The structural domains of the amino acid sequence of this protein were predicted by SMART online software, and the results showed that there is a Pfam:DUF3446 structural domain between amino acids 137 and 220, respectively, and a Pfam:DUF3432 structural domain between amino acids 452 and 530, respectively, and that the EGR1 protein structure also contains three low-complexity regions and three ZnF_C2H2 (Figure 2H)

### 3.3. Analysis of EGR1 Expression Profiles in Different Tissues

**Figure 3:**
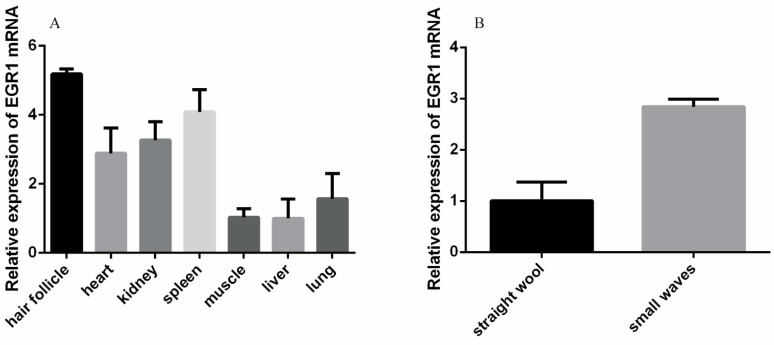
Expression level analysis of *EGR1*. (**A**) Expression profiles of *EGR1* in different tissues. (**B**) Expression of the *EGR1* gene in different patterns.

According to RT-qPCR results, the *EGR1* gene was widely expressed in the heart, liver, spleen, lung, kidney, muscle, and hair follicle tissues of Hu sheep. The highest expression was observed in hair follicle tissue, followed by spleen, and the least in the liver. *EGR1* gene expression in different patterns of Hu sheep was higher in small waves than in straight wool (Figure 3).

### 3.4. EGR1 Promoted Proliferation of DPCs

**Figure 4:**
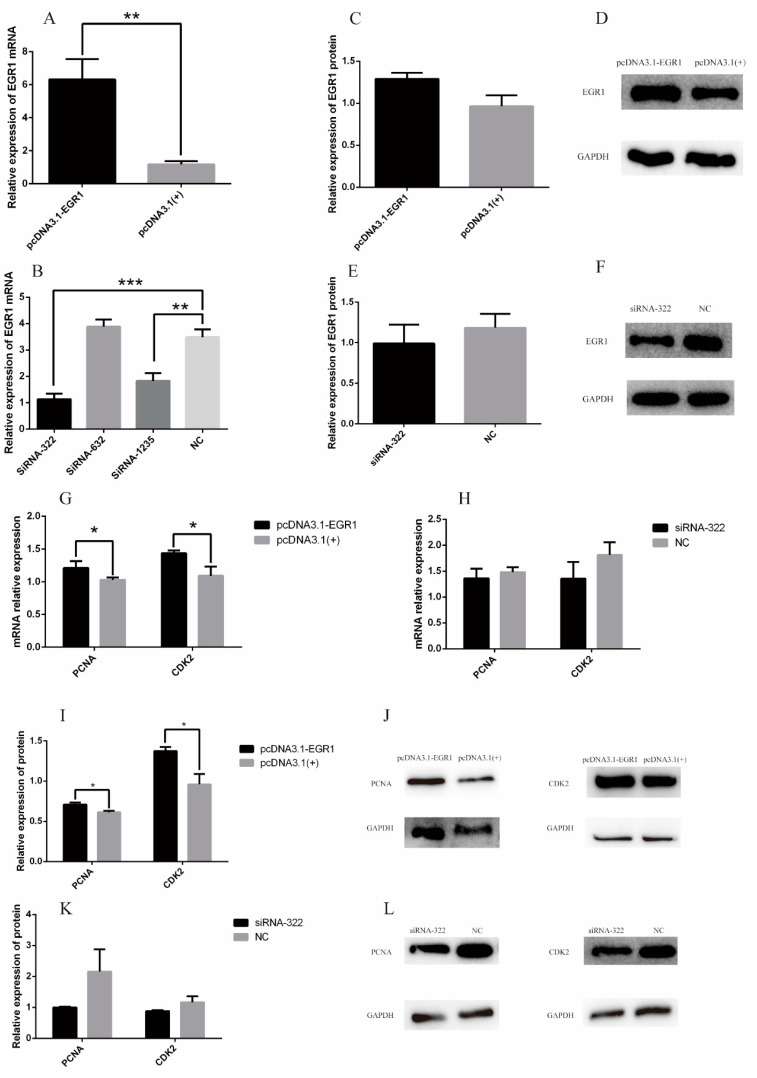
The effects of *EGR1* on dermal papilla cell proliferation. (**A**) mRNA relative expression of *EGR1* after pcDNA3.1-EGR1 transfection. (**B**) Relative expression of mRNA for *EGR1* in DPCs transfected with siRNA. (**C**) Relative expression of EGR1 protein after pcDNA3.1-EGR1 transfection. (**D**) Protein bands after pcDNA3.1-EGR1 transfection. (**E**) Relative expression of protein for EGR1 gene in DPCs transfected with siRNA-322. (**F**) Protein bands for EGR1 in DPCs transfected with siRNA-322. (**G**) mRNA relative expression of *PCNA* and *CDK2* after pcDNA3.1-EGR1 transfection. (**H**) The mRNA expression level of *PCNA* and *CDK2* in DPCs transfected with siRNA-322. (**I**) The protein expression level of PCNA and CDK2 after pcDNA3.1-EGR1 transfection. (**J**) Protein bands for PCNA and CDK2 after pcDNA3.1-EGR1 transfection. (**K**) Relative expression of protein for PCNA and CDK2 in DPCs transfected with siRNA-322. (**L**) Protein bands for PCNA and CDK2 in DPCs transfected with siRNA-322. “*” represents a significant difference (*p* < 0.05), “**” represents a highly significant difference (*p* < 0.01), “***” represents an extremely significant difference (*p* < 0.001).

To verify the effect of *EGR1* on DPCs, siRNA-322, siRNA632, siRNA1235, NC, pcDNA3.1-EGR1, and pcDNA3.1(+) were introduced into DPCs, cultured in 12-well plates and 6-well plates, respectively. The experiments were grouped as follows: siRNA-322, siRNA-632, siRNA-1235 and NC were transfected with DPCs at the concentration of 50 nM, and each treatment was repeated three times to verify the effect of the three siRNAs. RT-qPCR results showed that pcDNA3.1-EGR1 could up-regulate *EGR1* expression at the mRNA expression level (*p* < 0.01) (Figure 4A). The expression of *EGR1* gene was significantly reduced after transfection with siRNA-322 (*p* < 0.001). *EGR1* mRNA expression was significantly reduced after transfection with siRNA-1235 (*p* < 0.01), while siRNA-632 had no inhibitory effect on *EGR1* expression in Hu sheep DPCs (Figure 4B). Therefore, we chose to transfect siRNA-322 into DPCs for the following Western blot, EDU and CCK8 experiments. Western blot results showed that pcDNA3.1-EGR1 up-regulated EGR1 protein expression (Figure 4C,D), whereas siRNA-322 down-regulated EGR1 protein expression (Figure 4E,F). In addition, markers associated with cell proliferation, including PCNA and CDK2, were significantly increased at the mRNA level after pcDNA3.1-EGR1 transfection, whereas they were decreased at the mRNA level after siRNA-322 transfection (Figure 4G,H). Western blot results showed that markers associated with cell proliferation, including PCNA and CDK2, were significantly increased following pcDNA3.1-EGR1 transfection (Figure 4I,J) and decreased under siRNA-322 treatment (Figure 4K,L).

**Figure 5:**
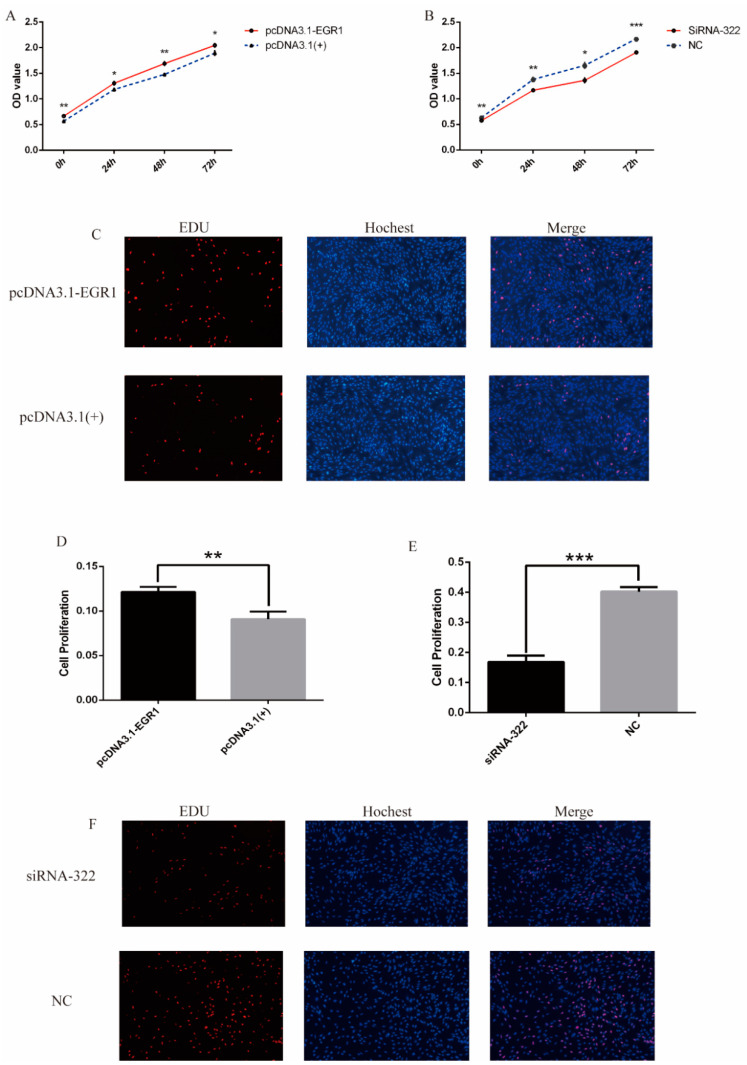
Effect of *EGR1* on the proliferation of DPCs. (**A**) The dermal papilla cell activity after overexpression of *EGR1* by CCK-8. (**B**) The dermal papilla cell activity after *EGR1* down-regulation by CCK-8. (**C**,**D**) The proliferation rate of DPCs after overexpression of *EGR1* by EDU immunofluorescence (100×). (**E**,**F**) The rate of proliferating cells after *EGR1* down-regulation (100×). “*” represents a significant difference (*p* < 0.05), “**” represents a highly significant difference (*p* < 0.01), “***” represents an extremely significant difference (*p* < 0.001).

To evaluate the proliferative effect of *EGR1* on DPCs, cell proliferation was detected. CCK-8 assay revealed that the proliferation rate of cells in the pcDNA3.1-EGR1 transfected group was significantly higher than that in NC (Figure 5A), while the proliferation rate under siRNA322 treatment was significantly lower than that in NC (Figure 5B). The EDU assay demonstrates similar results, with *EGR1* playing a positive effect on the proliferation of DPCs (Figure 5C–F).

## 4. Discussion

The lambskin quality is majorly determined by the pattern of lambskin; however, in recent years, the lambskin market has been shrinking and its economic value has been decreasing, leading to the neglection of lambskin trait breeding selection and the quality being in decline. However, the lambskin trait, as a unique and excellent trait of Hu sheep, has an important value for germplasm conservation. Therefore, it is of great social value to uncover the molecular mechanism underlying lambskin pattern formation for the future use of molecular breeding approaches to protect and improve the quality of lambskin.

Lambskin pattern formation is closely related to wool bending, which is determined by the cellular activity of the hair follicle cells; precisely, the activity of DPCs and other hair follicle cells affects hair bending by direct or indirect means [5]. DPCs are a group of cells that differentiate from dermal mesenchymal cells and are located in the center of the hair follicle [17]. They play a leading role in the morphogenesis and cyclic regulation of the hair follicle, and are the regulatory center of hair follicle development [18,19]. The activity of the hair follicle depends on DPCs providing it with a number of important nutrients, as well as signal regulators [20,21]. DPCs are considered to be multifunctional stem cells that regulate the growth and development of hair follicles [22]. Some researchers believe that the main cause of hair follicle curvature is the autonomous functioning of multiple papillae centers formed in the dermal papillae, which probably causes asymmetric hair growth and hair curvature [5]. Others insist that curly hairs originate from a hair bulb surrounded by abundant proliferating cells that cause curved hair growth due to the uneven distribution of their proliferation space [23]. As DPCs have certain potential to proliferate and differentiate and are present in the center of the hair bulb, this study investigates the formation of curved wool growth from the perspective of DPCs proliferation.

*EGR1* is an important transcription factor that has been widely studied in the areas of oncology, neuropsychiatric disease and diabetic kidney disease (DKD) [24,25,26], but it is not clear whether *EGR1* is associated with hair growth. *EGR1* encodes a protein with a zinc-binding finger structure, which acts as a DNA-binding domain in several transcriptional regulatory proteins [27]. *EGR1* is abundantly expressed during embryogenesis in bone, tendons and skeletal muscle in mice [28]. Interestingly, mice lacking *EGR1* could survive despite a reduced body size, suggesting that *EGR1* may be associated with organismal development [29,30,31]. Collectively, *EGR1* mainly plays a positive regulatory role for cell growth and development, consistent with the basic characteristics of this gene; our results also showed that *EGR1* was able to promote the proliferation of dermal papilla cells. Additionally, *EGR1* can be regulated at the epigenetic level through microRNAs (miRNAs). Indeed, quite a lot of studies reported direct or indirect targeting of *EGR1* by miR-203a, miR-377-3p, miR-125b-2-3p and miR-301b in many cancer cell lines, which slowed down the proliferation of cancer cells to a certain extent by inhibiting the expression of *EGR1* [32,33,34,35]. In the investigation of transcription factors important for the *BMP7* promoter in Hu sheep, Lv et al. [14] found that *EGR1* is located in the core transcriptional region, and that its expression enhances the transcriptional regulation of *BMP7*, a candidate gene involved in promoting proliferation in Hu dermal papilla cells. In addition, Adly et al. [36] found that *BMP7* was highly expressed in hair follicles during the anagen phase and in epidermal hair papillae, and that when follicle cells proliferated at the basal level, these cells immediately expressed *BMP7* at high levels; moreover, as follicle cells proliferated into the regressive and quiescent phases, most follicle cells ceased to express *BMP7*, and expression in epidermal hair papillae diminished accordingly. Combining previous findings with the results of our study, we can speculate that *EGR1*, as an important transcription factor of *BMP7*, can enhance the expression of the *BMP7* gene and thus promote the proliferation of hair papilla cells. Meanwhile, in our study, we also identified that *EGR1* expression was higher in DPCs of Hu sheep small waves than in straight wool. The proliferation rate of DPCs was significantly increased after overexpression of *EGR1* in DPCs. The reports confirmed that the proliferation of dermal papilla cells led to curly hair growth; additionally, in our study, the curl was higher in the small waves group, in which the expression level of *EGR1* was also higher than that of the straight wool group, which further confirmed the positive correlation between the expression of *EGR1* and the proliferation of dermal papilla cells.

Numerous pathways have been proved to be involved in hair follicle growth and development, among which the MAPK/ERK pathway is a classical activating proliferative pathway. One study revealed that *EGR1* expression was significantly reduced after treatment with inhibitors of the MAPK/ERK pathway, suggesting that *EGR1* was closely related to the MAPK/ERK pathway [37]. In addition to this, it has been shown that attenuating the nuclear fraction of *EGR1* apparently inhibits the survival of breast cancer cells by inhibiting MAPK phosphorylation [38]. *EGR1* is an activator of the MAPK/ERK pathway, while the activated pathway, in turn, enhances *EGR1* expression. Upregulated *EGR1* promotes cell proliferation and continues to enhance activation of the MAPK/ERK pathway, which is a key pathway regulating the biological activity of the hair follicle through a positive feedback loop [39]. Therefore, it is reasonable to infer that *EGR1* may promote hair follicle growth and development by enhancing the MAPK/ERK pathway, which is also the direction of our subsequent study.

## 5. Conclusions

We cloned the sequence of CDS region of *EGR1* in Hu sheep and found that it can promote the proliferation of dermal papilla cells after cellular analysis, which provides a certain basis for the subsequent study of the mechanism of lambskin pattern formation in Hu sheep.

## Figures and Tables

**Table 1:** The sequence information of siRNA-BMP4.

Name	Sequence (5′→3′)
siRNA-322	CCUGACAUCUCUCUGAAUATT UAUUCAGAGAGAUGUCAGGTT
siRNA-632	CCACACCUAACACUGACAUTT AUGUCAGUGUUAGGUGUGGTT
siRNA-1235	GCAAGAGGCAUACCAAGAUTT AUCUUGGUAUGCCUCUUGCTT

**Table 2:** The sequence information of genes for RT-qPCR.

Gene ID	Sequences (5′→30′)	Product Length/bp	Accession No.
*EGR1*	F: TTCAACCCTCAGGGGGAGG R: CGCTGACCAGACTGAAGAGG	223	EU552504.1
*PCNA*	F: TCTGCAAGTGGAGAACTTGGAA R: AGGAGACAGTGGAGTGGCTT	162	XM_004014340.5
*CDK2*	F: TGGGCCAGGCAGGATTTTAG R: GTCGAAGGTGAGGTACTGGC	166	FJ422550.1
*GAPDH*	F: TCTCAAGGGCATTCTAGGCTAC R: GCCGAATTCATTGTCGTACCAG	151	NM_001190390.1

**Table 3:** Similarity analysis of nucleotide and amino acid of EGR1.

Species	GenBank No.	Nucleotide (%)	Amino Acid (%)
*Bos taurus*	AY924307.1	97.06	98.53
*Homo sapiens*	MK681487.1	92.03	95.88
*Rattus norvegicus*	AY551092.1	84.85	85.69
*Mus musculus*	BC138615.1	84.70	86.42

## Data Availability

The raw data supporting the conclusions of this article will be made available by the authors, without undue reservation.
